# Single administration of Selective Internal Radiation Therapy versus continuous treatment with sorafeNIB in locally advanced hepatocellular carcinoma (SIR*ve*NIB): study protocol for a phase iii randomized controlled trial

**DOI:** 10.1186/s12885-016-2868-y

**Published:** 2016-11-07

**Authors:** Mihir Gandhi, Su Pin Choo, Choon Hua Thng, Say Beng Tan, Albert Su Chong Low, Peng Chung Cheow, Anthony Soon Whatt Goh, Kiang Hiong Tay, Richard Hoau Gong Lo, Brian Kim Poh Goh, Jen San Wong, David Chee Eng Ng, Khee Chee Soo, Wei Ming Liew, Pierce K. H. Chow, on behalf of Asia-Pacific Hepatocellular Carcinoma Trials Group

**Affiliations:** 1 Biostatistics, Singapore Clinical Research Institute, #02-01, Nanos, 31 Biopolis Way, Singapore, Singapore; 2 Centre for Quantitative Medicine, Duke-NUS Medical School, 8 College Road, Singapore, Singapore; 3 Tampere Center for Child Health Research, University of Tampere and Tampere University Hospital, Tempere, Finland; 4 Division of Medical Oncology, National Cancer Centre Singapore, 11 Hospital Drive, Singapore, Singapore; 5 Division of Oncologic Imaging, National Cancer Centre Singapore, 11 Hospital Drive, Singapore, Singapore; 6 Office of Research, Singapore Health Services, 31 Third Hospital Avenue, #03-03 Bowyer Block C, Singapore, Singapore; 7 Clinical Sciences, Duke-NUS Medical School, 8 College Road, Singapore, Singapore; 8 Department of Diagnostic Radiology, Singapore General Hospital, Outram Road, Singapore, Singapore; 9 Department of Hepato-pancreato-biliary and Transplant Surgery, Singapore General Hospital, Outram Road, Singapore, Singapore; 10 Department of Nuclear Medicine and PET, Singapore General Hospital, Outram Road, Singapore, Singapore; 11 Division of Surgical Oncology, National Cancer Centre Singapore, 11 Hospital Drive, Singapore, Singapore; 12 Project Management, Singapore Clinical Research Institute, #02-01, Nanos, 31 Biopolis Way, Singapore, Singapore; 13 Office of Clinical, Academic and Faculty Affairs, Duke-NUS Medical School, 8 College Road Singapore, Singapore; 14 Program in Translational and Clinical Liver Research, National Cancer Centre Singapore, Singapore, Singapore

**Keywords:** Advanced hepatocellular carcinoma, Liver cancer, Radioembolisation, Selective internal radiation therapy, SIR-Spheres, Sorafenib, Systemic therapy, Asia-Pacific, Randomized controlled trial, Phase III

## Abstract

**Background:**

Approximately 20 % of hepatocellular carcinoma (HCC) patients diagnosed in the early stages may benefit from potentially curative ablative therapies such as surgical resection, transplantation or radiofrequency ablation. For patients not eligible for such options, prognosis is poor. Sorafenib and Selective Internal Radiation Therapy (SIRT) are clinically proven treatment options in patients with unresectable HCC, and this study aims to assess overall survival following either SIRT or Sorafenib therapy for locally advanced HCC patients.

**Methods:**

This investigator-initiated, multi-centre, open-label, randomized, controlled trial will enrol 360 patients with locally advanced HCC, as defined by Barcelona Clinic Liver Cancer stage B or stage C, without distant metastases, and which is not amenable to immediate curative treatment. Exclusion criteria include previous systemic therapy, metastatic disease, complete occlusion of the main portal vein, or a Child-Pugh score of >7. Eligible patients will be randomised 1:1 and stratified by centre and presence or absence of portal vein thrombosis to receive either a single administration of SIRT using yttrium-90 resin microspheres (SIR-Spheres®, Sirtex Medical Limited, Sydney, Australia) targeted at HCC in the liver by the trans-arterial route or continuous oral Sorafenib (Nexavar®, Bayer Pharma AG, Berlin, Germany) at a dose of 400 mg twice daily until disease progression, no further response, complete regression or unacceptable toxicity. Patients for both the Sorafenib and SIRT arms will be followed-up every 4 weeks for the first 3 months and 12 weekly thereafter. Overall survival is the primary endpoint, assessed for the intention-to-treat population. Secondary endpoints are tumour response rate, time-to-tumour progression, progression free survival, quality of life and down-staging to receive potentially curative therapy.

**Discussion:**

Definitive data comparing these two therapies will help to determine clinical practice in the large group of patients with locally advanced HCC and improve outcomes for such patients.

**Trial registration:**

ClinicalTrials.gov identifier, NCT01135056, first received 24, May 2010.

## Background

The incidence and prevalence of hepatocellular carcinoma (HCC) is highly variable in different regions of the world, but the burden is predicted to increase in future years [1]. Approximately 70–80 % of all cases of HCC occur in Asia where it is an important public health concern [2]. Although around 20 % of patients diagnosed with early stage HCC may benefit from potentially curative ablative therapies, such as surgical resection, liver transplantation or radiofrequency ablation [3–5], most patients are diagnosed at an intermediate to advanced stage of HCC, when treatment options are limited and the prognosis poor [6, 7]. In patients with untreated advanced HCC median survival time is approximately 5–7 months, although this varies depending on the Child‐Pugh score [8–10].

The only systemic therapy shown to confer survival advantage in patients with unresectable advanced HCC is Sorafenib (Nexavar®, Bayer Pharma AG, Berlin, Germany) [11–13]. In the pivotal Sorafenib Hepatocellular Carcinoma Assessment Randomized Protocol (SHARP) trial in patients with advanced HCC, Sorafenib treatment significantly increased median overall survival (OS) by 2.8 months versus placebo (10.7 months versus 7.9 months, respectively; *p* < 0.001) [6]. A subsequent randomised controlled trial in the Asia-Pacific region confirmed these findings, showing median OS of 6.5 months with Sorafenib treatment versus 4.2 months with placebo (*p* < 0.014) [13]. As a result of these data, Sorafenib is the current recommended first-line treatment for advanced (Barcelona Clinic Liver Cancer [BCLC] stage C) HCC [2]. Effective systemic therapy is an important option for the treatment of HCC in the subset of patients with extrahepatic metastases and Sorafenib resulted in a median OS of 5.7 months in this group [14]. However, the relative benefits of Sorafenib and loco-regional ablative therapy are unclear in the larger group of patients with locally advanced HCC. This is of critical importance since HCC is a fast-growing locally aggressive disease frequently leading to the patient’s death before extrahepatic metastases have developed. Consequently, the response of loco-regional disease to first-line therapy determines survival in these patients.

Selective Internal Radiation Therapy (SIRT) with yttrium-90 (Y-90) resin microspheres (SIR-Spheres®; Sirtex Medical Limited, Sydney, Australia), is one potential alternative treatment for locally advanced HCC. SIRT enables targeted delivery of radiation to the tumours, while largely sparing the surrounding liver parenchyma. A meta‐analysis showed a high response rate to Y-90 SIRT in HCC patients [15]. Population disparity prevented assessment of OS in this meta-analysis, but cohort studies of patients with HCC receiving SIRT report median OS between 7.0 and 26.3 months [16–25].

Small-scale, retrospective studies have compared SIRT with Y-90 resin microspheres and Sorafenib, and suggest similar median OS in patients with BCLC stage B or C disease [24, 26, 27], and there are some indications that in patients with locally advanced HCC and portal vein thrombosis (PVT), SIRT is particularly beneficial [14, 22, 27–29].

However, larger-scale studies are needed to assess this comparison, and therefore, the Selective Internal Radiation Therapy versus sorafeNIB (SIR*ve*NIB) trial has been designed as a prospective, randomized, open-label, multicentre trial to compare median OS in patients with locally advanced HCC receiving either SIRT with Y-90 resin microspheres or Sorafenib.

Another large-scale comparative study of similar design in patients with advanced HCC, SorAfenib versus Radioembolisation in Advanced Hepatocellular carcinoma (SARAH), is ongoing in Europe [30], and these two studies could potentially be combined in a future meta-analysis.

## Methods/Design

### Ethics

The SIR*ve*NIB trial will be conducted in accordance with the Declaration of Helsinki and current Good Clinical Practice guidelines, and all participating centres will have obtained the relevant ethics committee approval before patient enrolment.

### Eligible population

The inclusion and exclusion criteria for the SIR*ve*NIB trial are summarised in Table 1. Informed consent will be obtained from each participant.

**Table 1:** Patient eligibility criteria for SIR*ve*NIB trial

Inclusion criteria	Exclusion criteria
• Unequivocal diganosis of locally advanced HCC without extrahepatic metastases • Written informed consent provided • Aged ≥18 years • Patients with HCC that is not amenable to surgical resection, immediate liver transplantation, or that could be treated with local ablative techniques (e.g. radiofrequency ablation) • Locally advanced HCC as defined by BCLC (B) intermediate stage or BCLC (C) advanced stage • At least one lesion that can be accurately measured in at least one dimension (longest diameter to be recorded) as ≥10 mm with spiral CT scan or MRI • ECOG performance status 0–1 • Adequate haematological function: haemoglobin ≥9.5 g/dl, leukocytes ≥2500/mm^3^, platelets ≥80,000/mm^3^, INR ≤2.0 • Adequate kidney function: creatinine <2.0 mg/dl • Adequate hepatic function: albumin ≥2.5 g/dl, bilirubin ≤2 mg/dl; ALP, AST or ALT ≤5 x ULN • Liver cirrhosis Child-Pugh A–B (up to 7 points) • Life expectancy of at least 3 months without active treatment	• Patients who have had >2 administrations of hepatic artery directed therapy • Hepatic artery directed therapy <4 weeks before study entry • Systemic chemotherapy for HCC, except previous adjuvant or neoadjuvant therapy given >6 months before enrollment • Previous treatment with Sorafenib or VEGF inhibitors • Previous radiotherapy for HCC or other malignancy • Intractable ascites, or other clinical signs of liver failure • Complete thrombosis of the main portal vein • Extrahepatic metastases, except lung nodules <1 cm or local-regional lymph nodes <2 cm in greatest diameter • Clinical signs of central nervous system metastases • Other concurrent malignancy, except for adequately treated basal cell or squamous cell skin cancer, in situ cervical cancer, or other cancer for which the patient has been disease free for ≥5 years • Uncontrolled intercurrent illness • Contraindications angiograghy to hepatic artery catheterisation: severe peripheral arterial disease precluding catheterisation, bleeding diathesis not correctable by standard forms of therapy, portal hypertension with hepato-fugal flow • History of allergy to SIR-spheres, Soranefib or related agents • Patient unable or unwilling to understand or sign the written informed consent • Currently enrolled in another investigational therapeutic drug or device study • Women, unless postmenopausal or surgically sterile are unwilling to practice effective contraception • Men unwilling to use effective contraception during the course of the study

*ALP* alkaline phosphatase, *ALT* alanine aminotransferase, *AST* aspartate transaminase, *CT* computed tomography, *MRI* Magnetic resonance imaging, *ECOG* Eastern Cooperative Oncology Group, *HCC* hepatocellular carcinoma, *INR* international normalised ratio, *ULN* upper limit of normal

### Overview of trial design

SIR*ve*NIB is a prospective, randomized open-label, multicentre trial comparing SIRT and Sorafenib in patients with locally advanced HCC. In SIR*ve*NIB, the aim will be to recruit a minimum of 360 patients from a minimum of 15 sites across the Asia-Pacific region.

**Fig. 1:**
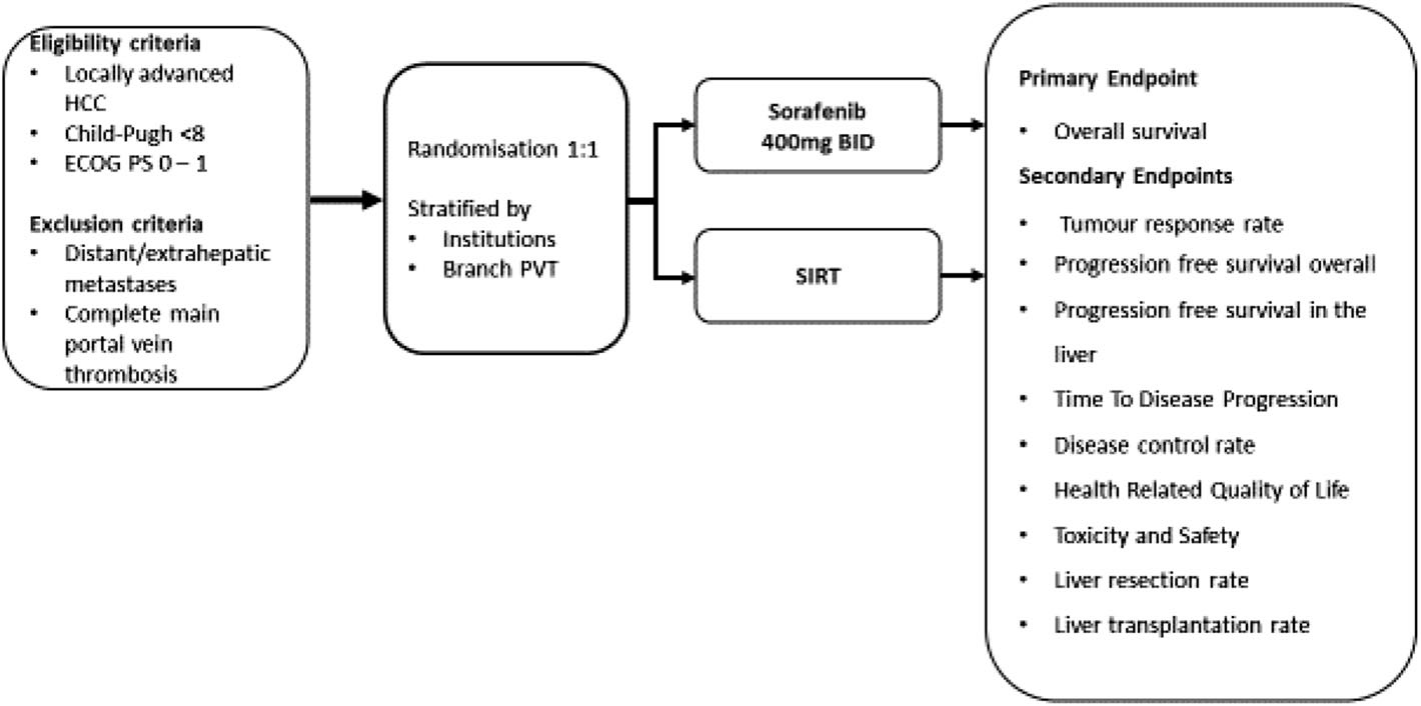
Overview of the SIR*ve*NIB trial design. ECOG, Eastern Cooperative Oncology Group; PVT, portal vein thrombosis

Eligible patients will be randomized 1:1 to receive either systemic therapy with oral Sorafenib or a session of SIRT with SIR-Spheres® (Fig. 1). Randomisation will be stratified by centre and the absence or presence of PVT.

### Treatment

Treatment in the Sorafenib arm will commence within 1 week after randomisation. Patients will receive oral Sorafenib, 400 mg twice daily. Sorafenib treatment will be continued until there is evidence of treatment failure (lack of efficacy resulting in tumour progression at any site determined by CT or MRI scan); there is a cure or complete response and/or the patient undergoes surgical resection, liver transplantation or ablative therapy; unacceptable toxicity occurs; or the patient requests an end to treatment. As published previously, doses may be delayed and/or reduced for clinically significant haematological toxicities and other toxicities or adverse events (AEs) related to study therapy. Dose reductions first to 400 mg/day and then to 400 mg every second day will be allowed, and if further dose reductions are required the patient should be discontinued. For non-haematological AEs other than skin toxicity, treatment will be interrupted for any grade 3 AE, and the dose subsequently reduced by one level. For skin toxicity, treatment will be interrupted for any grade 2 or grade 3 AE and a decreased dose frequency or level will be subsequently considered. The dose may be re-escalated once the toxicities or AEs have resolved [31].

Patients randomised to SIRT will receive SIR-Spheres at the patient-specific prescribed activity within 35 days after signing of informed consent form, and after the baseline assessment of their suitability for the procedure. The assessment comprises a hepatic angiogram, and a liver-to-lung shunt pre-assessment with Technetium-99 m (^99m^Tc)-labelled human serum albumin. The hepatic angiogram will determine the vascular anatomy of the liver in order to plan the optimal delivery of the SIR-Spheres. The ^99m^Tc lung-shunt study will assess the presence and degree of lung shunting from the liver. Patients randomised to SIRT, but who are found to be unsuitable for treatment will be included in the SIRT intention to treat analysis. The prescribed activity of SIR-Spheres® will be calculated based on the patient’s body surface area (BSA) model [32], or the partition model [33]. If the BSA method is used for dose calculation and the percentage lung shunting exceeds 20 % of the hepatic artery blood flow, as determined by ^99m^ Tc-scan, the partition model may be used to adjust the prescribed activity so that the radiation absorbed dose to the lungs does not exceed 20 Gy.

### Assessments

A quadriphasic contrast-enhanced spiral CT scan of the abdomen/pelvis will be performed at screening to diagnose HCC according to the American Association for the Study of Liver Diseases (AASLD) criteria [34], determine the extent of liver disease and to exclude extra-hepatic abdominal or pelvic metastases. A biopsy positive for HCC is required for diagnosis if the tumour does not fulfil the AASLD radiological criteria. A thoracic CT scan will be performed to exclude lung metastases. MRI scans will be used in lieu of CT scans in patients for whom CT scanning is not clinically feasible. Each of these CT series will be performed less than 28 days before informed consent is received. All radiology images in this trial will be centrally reviewed by treatment-blinded radiologists at the National Cancer Center Singapore.

All patients will be assessed by the schedule summarised in Table 2. Assessments are at 4-week intervals for the first three months, and then 12-week intervals thereafter. After study conclusion patients will be followed for survival or death at 12-week intervals.

**Table 2:** SIRveNIB trial assessment schedule

Schedule	Screening/Baseline (Eligibility) Randomisation^a^	During Protocol Therapy					Study Conclusion	Post Study Conclusion Follow-Up
		Week 2^b^	Week 4	Week 8	Week 12	12-weekly thereafter	As appropriate^c^	12 weekly
Informed consent	X							
Demographics	X							
Medical and surgical history	X							
Concurrent illness	X							
Concomitant medications	X^d^	X^d^	X^d^	X^d^	X^d^	X^d^	X^d^	
Clinical assessment & physical examination • Height (Baseline only) • Weight • Blood pressure • Body temperature	X		X	X	X	X	X	
Performance status • ECOG	X		X	X	X	X	X	
Haematology • Leukocytes • Platelets • Haemoglobin • INR	X		X	X	X	X	X	
Hepatitis serology • Hep Bsag • Anti-HCV IgG • Hep B Core Antibody IgG (optional)	X^e^							
Renal function • Creatinine	X		X	X	X	X	X	
Liver function • AST/ALT • ALP • Total bilirubin • Albumin	X		X	X	X	X	X	
Pregnancy test (as appropriate)	X^f^							
Tumour marker • Serum AFP	X				X	X	X	
EQ-5D HRQoL	X		X	X	X	X	X	X
CT or MRI scan: chest/abdomen/pelvis^h, i^	X				X	X		
*SIRT-arm ONLY* • Hepatic angiogram • ^99m^Tc- MAA lung shunt study	X ^g^							
Response assessment					X	X	X	
*Sorafenib arm ONLY* • Toxicity assessment • Dose delay/modification		X^b^	X	X	X	X	X	
AE/SAE	AE/SAE for the Sorafenib arm will be recorded from the time of signing the ICF until 30 days after the final dose of Sorafenib, or until commencement of the next alternative therapy, whichever is earlier. AE/SAE for the SIRT arm will be recorded from the time of signing the ICF until 30 days post-SIRT regardless of causality and for a further 5 months thereafter if judged by the investigator to be causally related to SIRT or Sir-Spheres, or until commencement of the next alternative therapy, whichever is earlier. If the AE/SAE is a Sorafenib or SIRT related toxicity follow-up will continue until resolution.							
Survival								X

^a^Screening assessments performed within 28 days before signing of informed consent can be used to confirm eligibility

^b^Sorafenib arm only. Sorafenib patients contacted at Week 2 to assess treatment related toxicity and interrupt/modify the dose as necessary

^c^Disease progression, death, complete regression, unacceptable toxicity, patient responds to treatment and becomes eligible for surgical resection, liver transplantation or ablative therapy, lost to follow-up, patient’s request for withdrawal

^d^Concomitant medication to be recorded from screening up to 30 days post-study conclusion, or until commencement of the next alternative therapy, whichever is earlier

^e^If either the Hepatitis B Surface Antigen (Hep Bsag) test or anti-HCV IgG test is positive, the other test will be optional. Hepatitis B Core Antibody IgG test is optional

^f^Women of reproductive potential must have a negative pregnancy test before commencing treatment. Test to be repeated if pregnancy is suspected during the study

^g^Hepatic angiogram and ^99m^Tc-MAA lung shunt study to be performed after randomisation and prior to treatment commencement only for SIRT arm group

^h^The same radiological assessment method must be used throughout the study

^i^ Assessment for tumour response rate to be done every 12 weeks from date of randomisation until first evidence of disease progression

Study conclusion is defined as disease progression, death, complete regression, unacceptable toxicity, patient-undergoing surgical resection, liver transplantation or ablative therapy due to a sufficient response with therapy, loss to follow-up, or patient’s request for withdrawal.

### Outcome measures

The primary endpoint of the SIR*ve*NIB trial is overall survival (OS). Secondary endpoints include: time to progression (TTP), progression-free survival (PFS), overall and in the liver; tumour response rate (assessed by Response Evaluation Criteria in Solid Tumours [RECIST] version 1.1) [35]; disease control rate; health-related quality of life (HRQoL); safety and toxicity; and liver resection rate and liver transplantation rate.

### Outcome definitions

- OS – the time from the date of randomisation to death from any cause.
- TTP – the time from the date of randomisation to tumour progression at any site in the body.
- PFS at any site – time from the date of randomisation to tumour progression at any site in the body or death, whichever is earlier.
- PFS in the liver –time from the date of randomisation to tumour progression in the liver or death whichever is earlier.
- Tumour response rate is the number of patients whose best overall response rate (best tumour response over the whole study between randomisation and the last tumour assessment) is partial response (PR) or complete response (CR), divided by the total number of patients in the analysis population.
- Disease control rate – the number of patients whose best overall response is PR, CR or stable disease (SD), divided by the total number of patients in the analysis population.
- HRQoL assessed using the EQ-5D questionnaire.
- Adverse events will be reported according to National Cancer Institute criteria (National Cancer Institute Common Terminology criteria for Adverse Events [NCI CTCAE] Version 4.02) [36].

### Sample size calculation and statistical considerations

Based on OS data reported by Kang et al. 2008 and by Sangro et al. 2010 [14, 37], in patients with locally advanced HCC median survival times of 9.35 months for Sorafenib-treated patients and 14 months for patients treated with Y-90 microspheres are assumed. This represents the minimum clinically meaningful difference to be detected.

Group-sequential methods are used to determine the sample size and study duration. Two interim analyses and a final analysis are planned to occur at equally spaced intervals after one-third, two-thirds and all of the planned number of deaths (events) have been reported. The planned sample size is determined assuming the use of a 2-sided log rank test with type I error of 0.05 and statistical power of 90 %. A dropout rate of up to 20 % is also factored into the computations. The anticipated study duration is 5 years; with 3 years of accrual and 2 years follow-up. This corresponds to an estimated hazard rate of 0.67 with an expected 266 deaths at the end of the study

The number of patients required for randomisation to detect a clinically relevant difference in OS time with SIRT versus Sorafenib was determined to be 360 patients (180 patients in each treatment arm).

### Interim analyses

Interim analyses for efficacy are planned after 33 and 65 % of the information (corresponding to 87/266 and 174/266 deaths) have been observed and using a critical *p*-value of *p* ≤ 0.0001 for the first analysis and *p* ≤ 0.015 for the second analysis These boundaries are obtained using the method of Lan and DeMets [38] and based on an overall significance level of 5 %. The final analysis will be performed after 266 reported deaths.

An independent Data Monitoring Committee will review the safety of the study and the results of the interim analyses and make appropriate recommendations to the trial steering committee. .

### Statistical analyses

All analyses will be performed using the intention-to-treat (ITT) principle where patients will be analysed according to their randomised group. The analysis of the primary outcome (OS) will be an unadjusted log rank test used to test and a proportional hazards model will be use to estimate the hazard ratio together with the corresponding 95 % confidence intervals (CI). Time to event curves (for OS and PFS) will be displayed using the method of Kaplan-Meier.

The tumour response rate, disease control rate and the rate of down-staging to surgical resection, radiofrequency ablation or liver transplantation compared between treatments using appropriate tests for proportions

HRQoL will be analysed using generalised estimating equations assuming a common (exchangeable/compound symmetric) correlation structure, following the guidelines of the EQ-5D questionnaire.

A Landmark analysis will be performed at 2-months post-randomization comparing patients classified into four groups: Those randomised to Sorafenib and (i) but did not receive 80 % of scheduled dose since randomization; (ii) those receiving at least 80 % of the scheduled dose; and, those randomized to SIRT therapy and (iii) did not receive the therapy; (iv) those who receiving the SIRT therapy within the first two months of randomization [39]. This analysis will be repeated using 40 and 60 % scheduled doses since randomization for defining groups (i) and (ii). These comparisons will be performed for the primary and secondary outcomes. Patients who died or discontinued the study before 2-months of randomization will not be included from the analysis. Additional sub-group analyses will be performed based on patients’ baseline characteristics such as presence or absence of portal vein thrombosis, BCLC stage, prior HCC treatment, hepatitis status, ECOG performance status, tumour size (≤50 % of liver, > 50 % of liver), age (<65 years, ≥65 year), and gender.

As the SIRT therapy is a local treatment, treatment effect of progression in the liver as the first event will be investigated using a competing risk analysis. In this analysis death or progression outside the liver as the first event will be considered as a competing risk for liver progression. The method of Gray [40] will be used to compare groups with hazard ratios and 95 % CI estimated from the proportional hazards approach detailed by Fine and Gray [41]. Time to liver progression in the two groups will be displayed using cumulative incidence curves.

Toxicity will be reported according to NCI CTCAE Version 4.02 criteria.

## Discussion

The SIR*ve*NIB trial will compare the efficacy and safety of SIRT with yttrium-90 resin microspheres with that of Sorafenib in the treatment of locally advanced HCC. To the authors’ knowledge, no prospective, randomized controlled trials have been published comparing these treatment modalities in this patient population. Another study of similar design is ongoing in a European population, where the aetiology of HCC is different from that of the Asia-Pacific [30].

Sorafenib has been chosen as the control in the SIR*ve*NIB trial as the current most effective systemic treatment for patients with unresectable advanced HCC, SIRT with resin microspheres has also demonstrated efficacy in advanced HCC [14, 22, 28, 42, 43].

In the SIR*ve*NIB trial, OS has been chosen as the primary endpoint as it is a more robust measure than PFS, and the SHARP trial demonstrated the efficacy of Sorafenib based on this criterion. Moreover, the kinetics of tumour progression, as assessed from imaging techniques, is different between SIRT and Sorafenib, rendering OS the best option for comparison between arms in this trial. A European study with similar inclusion criteria is currently ongoing that compares Sorafenib with SIRT in patients with advanced HCC [30], and could be used for meta-analysis in the future.

In addition to efficacy analyses, the SIR*ve*NIB trial enables comparison of the toxicity caused by Sorafenib versus SIRT in patients with locally advanced HCC. This is important as the cardiovascular toxicity of Sorafenib has been highlighted in a meta-analysis of cancer patients (predominantly renal carcinoma) [44], but was not a common complication in the SHARP study [12].

Limitations to the SIR*ve*NIB study design have been addressed where feasible. While blinding is not possible due to the treatment methods, the potential biases caused by the lack of blinding have been minimised by the choice of OS as a robust primary endpoint.

The results from the SIR*ve*NIB trial will impact clinical practice. A definitive randomised controlled trial comparing the two most promising therapies in locally-advanced HCC should help determine the optimal treatment modality in this indication, or may help identify populations that are best suited to either therapy. In addition, the data generated from this study will determine the role of SIRT in in future consensus guidelines. The study will also pave the way for future trials in combined modality therapies in HCC.

### Trial status

The SIR*ve*NIB trial is currently ongoing. Patient recruitment closed on 25 May 2016 with a total of 360 participants randomised. The final analysis will be triggered after mortality reaches 266.

## Abbreviations

^99m^Tc: Technetium-99 m
AASLD: American Association for the Study of Liver Diseases
ALP: Alkaline phosphatase
ALT: Alanine aminotransferase
AST: Aspartate transaminase
BCLC: Barcelona clinic liver cancer
BOR: Best overall response
CI: Confidence interval
CR: Complete response
CT: Computed tomography
ECOG: Eastern Cooperative Oncology Group
HCC: Hepatocellular carcinoma
HRQoL: Health-related quality of life
INR: International normalised ratio
ITT: Intention to treat
MRI: Magnetic resonance imaging
NCI CTCAE: National Cancer Institute Common Terminology Criteria for Adverse Events
OS: Overall survival
PFS: Progression-free survival
PR: Partial response
PVT: Portal vein thrombosis
RECIST: Response Evaluation Criteria in Solid Tumours
SARAH: SorAfenib versus Radioembolisation in Advanced Hepatocellular carcinoma
SHARP: Sorafenib Hepatocellular carcinoma Assessment Randomized Protocol
SIRT: Selective Internal Radiation Therapy
SIRveNIB: Selective internal radiation therapy (SIRT) VErsus SorafeNIB in locally advanced hepatocellular carcinoma
TTP: Time to progression
ULN: Upper limit of normal
